# Effective treatment of SIVcpz-induced immunodeficiency in a captive western chimpanzee

**DOI:** 10.1186/s12977-017-0359-0

**Published:** 2017-06-02

**Authors:** Hannah J. Barbian, Raven Jackson-Jewett, Corrine S. Brown, Frederic Bibollet-Ruche, Gerald H. Learn, Timothy Decker, Edward F. Kreider, Yingying Li, Thomas N. Denny, Paul M. Sharp, George M. Shaw, Jeffrey Lifson, Edward P. Acosta, Michael S. Saag, Katharine J. Bar, Beatrice H. Hahn

**Affiliations:** 1 Department of Microbiology, Perelman School of Medicine, University of Pennsylvania, Philadelphia, PA USA; 2 Department of Medicine, Perelman School of Medicine, University of Pennsylvania, 409 Johnson Pavilion, 3610 Hamilton Walk, Philadelphia, PA 19104-6076 USA; 3 ChimpHaven, Inc., Keithville, LA USA; 4 Duke Human Vaccine Institute, Duke University School of Medicine, Durham, NC USA; 5 Institute of Evolutionary Biology, and Centre for Immunity, Infection and Evolution, University of Edinburgh, Edinburgh, UK; 6 AIDS and Cancer Virus Program, Leidos Biomedical Research, Inc., Frederick National Laboratory for Cancer Research, Frederick, MD USA; 7 Department of Medicine and Center for AIDS Research, University of Alabama at Birmingham, Birmingham, AL USA

**Keywords:** SIVcpz, Chimpanzees, Antiretroviral therapy, AIDS, Drug resistance

## Abstract

**Background:**

Simian immunodeficiency virus of chimpanzees (SIVcpz), the progenitor of human immunodeficiency virus type 1 (HIV-1), is associated with increased mortality and AIDS-like immunopathology in wild-living chimpanzees (*Pan troglodytes*). Surprisingly, however, similar findings have not been reported for chimpanzees experimentally infected with SIVcpz in captivity, raising questions about the intrinsic pathogenicity of this lentivirus.

**Findings:**

Here, we report progressive immunodeficiency and clinical disease in a captive western chimpanzee (*P. t. verus*) infected twenty years ago by intrarectal inoculation with an SIVcpz strain (ANT) from a wild-caught eastern chimpanzee (*P. t. schweinfurthii*). With sustained plasma viral loads of 10^5^ to 10^6^ RNA copies/ml for the past 15 years, this chimpanzee developed CD4+ T cell depletion (220 cells/μl), thrombocytopenia (90,000 platelets/μl), and persistent soft tissue infections refractory to antibacterial therapy. Combination antiretroviral therapy consisting of emtricitabine (FTC), tenofovir disoproxil fumarate (TDF), and dolutegravir (DTG) decreased plasma viremia to undetectable levels (<200 copies/ml), improved CD4+ T cell counts (509 cell/μl), and resulted in the rapid resolution of all soft tissue infections. However, initial lack of adherence and/or differences in pharmacokinetics led to low plasma drug concentrations, which resulted in transient rebound viremia and the emergence of FTC resistance mutations (M184V/I) identical to those observed in HIV-1 infected humans.

**Conclusions:**

These data demonstrate that SIVcpz can cause immunodeficiency and other hallmarks of AIDS in captive chimpanzees, including *P. t. verus* apes that are not naturally infected with this virus. Moreover, SIVcpz-associated immunodeficiency can be effectively treated with antiretroviral therapy, although sufficiently high plasma concentrations must be maintained to prevent the emergence of drug resistance. These findings extend a growing body of evidence documenting the immunopathogenicity of SIVcpz and suggest that experimentally infected chimpanzees may benefit from clinical monitoring and therapeutic intervention.

## Background

SIVcpz infection is common and widespread in two chimpanzee subspecies, the central chimpanzee (*P. t. troglodytes*) in west central Africa and the eastern chimpanzee (*P. t. schweinfurthii*) in the Democratic Republic of the Congo (DRC) and countries to the east [1–5]. Two other subspecies in west Africa (*P. t. verus*) and Nigeria/Cameroon (*P. t. ellioti*) are free of SIVcpz infection [3, 6, 7], because chimpanzees acquired this virus relatively more recently following the cross-species transmission and recombination of SIVs infecting monkeys on which they prey [8]. SIVcpz has been reported to have a negative impact on the health, reproduction and lifespan of chimpanzees living in the wild. Comprehensive natural history studies in Gombe National Park, Tanzania, revealed that SIVcpz-infected chimpanzees have a 10–16 fold increased risk of death and can develop CD4+ T cell depletion and AIDS-like immunopathology [9, 10]. Moreover, SIVcpz infected females were found to have lower birth rates and higher infant mortality compared to non-infected females [9], and one community with a high SIVcpz prevalence suffered a catastrophic population decline [11]. These results indicated that SIVcpz, unlike SIVs infecting other African primates such as sooty mangabeys (*Cercocebus atys*) or vervet monkeys (*Chlorocebus pygerythrus*), is pathogenic in its natural chimpanzee host [9–11].

*P. t. verus* apes comprise the great majority of captive chimpanzees worldwide, with 95% of founder animals in the United States having originated from West Africa [6, 12]. This explains the paucity of SIVcpz infections in captive populations, although a handful of SIVcpz infected *P. t. troglodytes* and *P. t. schweinfurthii* apes have been identified [13–16]. One such chimpanzee (Goran), who was rescued in Cameroon and studied in captivity for several years, developed CD4 T+ cell decline, severe thrombocytopenia, weight loss, and recurrent infections, and ultimately died of an AIDS-like illness [13]. Another chimpanzee (Noah), who was captured in the DRC and kept in a European primate center, also exhibited thrombocytopenia and some immunopathology [17]. Still another chimpanzee (Marilyn), who was wild-caught in west central Africa and utilized for biomedical research in the US, had normal laboratory and clinical findings, but died at age 26 after giving birth to still-born twins [14]. Finally, a number of bushmeat orphans identified in Gabon (GAB1 and GAB2) and Cameroon (CAM3, CAM4, CAM5, CAM13) were shown to be SIVcpz infected, but none of these could be studied prospectively since most died shortly after being rescued [15, 16]. Thus, only three naturally SIVcpz infected apes have been studied in captivity for an extended period of time.

Although most experimental lentivirus infections of chimpanzees were performed using HIV-1, a handful were infected with SIVcpz [17, 18]. Of six *P. t. verus* and two *P. t. schweinfurthii* apes exposed to SIVcpz by intra-venous, intra-rectal or intra-vaginal routes, six became productively infected [18]. Three of these subsequently died of cardiomyopathy, a frequent cause of death in captive chimpanzees unrelated to SIVcpz infection, while the other three remained clinically healthy for as many as 16 years. The seeming absence of disease progression in these animals prompted speculation that the increased mortality associated with SIVcpz in the wild might be due to factors other than, or in addition to, their lentiviral infection [17]. In addition, the possibility was raised that members of the *P. t. verus* subspecies, which are not naturally infected, may be more resistant to SIVcpz pathogenicity and/or that the SIVcpzANT strain used for these experimental infections was somehow attenuated [17]. Here, we studied one of these chimpanzees after his transfer from the Southwest National Primate Research Center to the Chimp Haven sanctuary, documenting disease progression and clinical immunodeficiency requiring antiretroviral therapy.

## Immunodeficiency in a western chimpanzee experimentally infected with SIVcpz

**Fig. 1:**
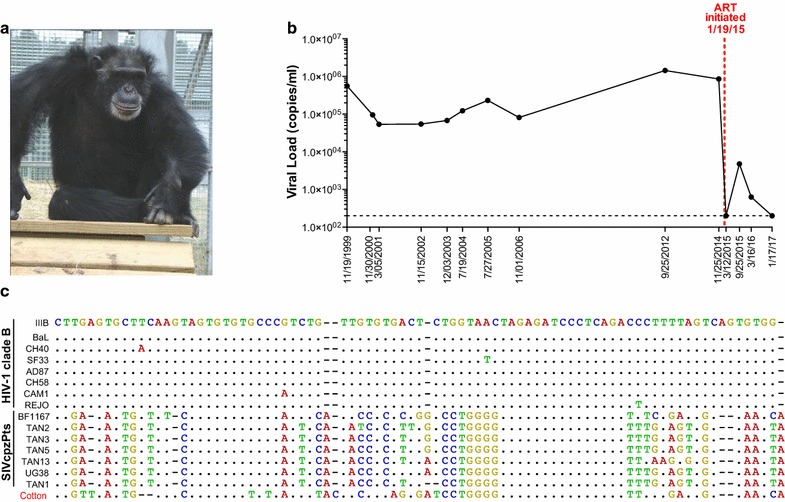
Virological evaluation of a western chimpanzee with long-term experimental SIVcpz infection. **a** Cotton (X115) after initiation of antiviral therapy. Cotton was infected with a highly divergent SIVcpz*Pts* strain (ANT) that differs from HIV-1 in up to 48% of Env protein sequences. **b** Plasma virus loads (copies/ml) in Cotton over a 17-year time span (sample dates are indicated). SIVcpzANT viral loads were determined using a sensitive validated RT-qPCR method that detects both SIVcpz and HIV-1 infections [21]. A *dashed red line* indicates the onset of antiretroviral therapy (January 19, 2015). **c** Nucleotide sequence alignment of HIV-1 clade B and SIVcpz*Pts* strains in the long terminal repeat (LTR) region (SIVcpzANT LTR sequences are not available). Sequences are compared to HIV-1/IIIb, with * dots* indicating sequence identity and *dashes* indicating gaps introduced for optimal alignment. LTR sequences from Cotton are much more closely related to SIVcpz*Pts* than to HIV-1 strains, indicating that he is solely infected with SIVcpzANT

Cotton is a 40-year old male chimpanzee (also termed X115; Fig. 1a) who was experimentally infected with SIVcpzANT in 1996 after an earlier exposure to HIV-1/IIIb [18]. The SIVcpz stock used for his infection was generated without interim in vitro culture by transferring peripheral blood mononuclear cells (PBMCs) from a naturally infected eastern chimpanzee (Noah, Ch-No) [19] to an uninfected cage mate (Niko; Ch-Ni), and then using plasma collected during the acute infection phase for intra-rectal inoculation of Cotton and others [18]. Immediately after SIVcpz infection, Cotton experienced CD4+ T cell depletion, which was followed by partial restoration and then gradual decline of CD4+ T cell levels over time [17]. Cotton arrived at the US sanctuary Chimp Haven in 2006 and CD4+ T cell counts determined in 2010 and 2014 showed 229 and 220 cells/μl, compared to ~1500 cells/μl before the infection [17]. Cotton also had low platelet counts of 101,000/μl and 90,000/μl in 2014 and 2015 (normal range 130,500–379,930/μl [20]), low albumin levels (2.0 g/dl, normal range 3.3–4.1 g/dl [20]), and persistent soft tissue infections. The latter were first noted in 2009 and included a necrotizing infection of the hand. In 2014, he developed purulent anal fistulas that persisted despite debridement and administration of multiple rounds of broad-spectrum antibiotics delivered orally and intravenously over the course of more than three months. The low CD4+ T cell counts, thrombocytopenia, hypoalbuminemia, and treatment-refractory soft tissue infections suggested that Cotton suffered from SIVcpz induced immunodeficiency.

To determine whether Cotton’s clinical symptoms were indeed a consequence of long-term SIVcpz infection, we determined his virus load in plasma samples taken as part of routine health examinations at Chimp Haven, as well as in a limited number of archived samples that were still available for years 1999–2006 (Fig. 1b). Since Cotton was exposed to HIV-1 prior to SIVcpzANT inoculation [18], we used a sensitive RT-qPCR method designed to amplify both HIV-1 and divergent SIVcpz strains by targeting a highly conserved region in the viral LTR [21]. This analysis revealed plasma viral titers ranging from 54,000 RNA copies/ml in 2001 to 1,441,000 RNA copies/ml in 2012 (Fig. 1b), significantly higher than previously reported for some of these same time points [17]. Since our RT-qPCR approach was rigorously validated using both human and chimpanzee plasma samples of known viral content, the previous results likely represent an underestimation of Cotton’s viral loads. To exclude a low-level HIV-1 infection, we sequenced the 121 base pair LTR region used for RT-qPCR analysis (Fig. 1c). Amplicons were prepared for MiSeq sequencing (Nextera DNA Library Prep Kit, Illumina, La Jolla, CA, USA) and the resulting reads were mapped to HIV-1 and SIVcpz*Pts* reference sequences, since LTR sequences of the original SIVcpzANT isolate are not available [22]. This approach yielded 11,679 paired-end reads, all of which were much more closely related to SIVcpz*Pts* than to HIV-1 sequences, including HIV-1/IIIb (Fig. 1c), indicating that Cotton is solely infected with SIVcpzANT.

## Antiretroviral therapy of SIVcpz-induced clinical immunodeficiency

Given Cotton’s laboratory and clinical findings, antiretroviral therapy (ART) was initiated on January 19, 2015, using a combination of the nucleoside reverse transcriptase inhibitors tenofovir disoproxil fumarate (TDF) and emtricitabine (FTC), and the integrase strand transfer inhibitor dolutegravir (DTG) at daily doses of 300, 200 and 50 mg, respectively. This regimen was selected because it is recommended for HIV-1 infected adults [23] and potently inhibits both HIV-1 and HIV-2 in humans [24–27]. It also potently inhibits SIVmac, a virus even more distantly related to HIV-1 than SIVcpzANT, in experimentally infected rhesus macaques [28]. As shown in Fig. 1b, this regimen reduced SIVcpzANT viremia in Cotton to undetectable levels as determined by RT-qPCR (less than 200 copies/ml) at the first blood analysis 7 weeks after initiation of therapy (3/12/15). The treatment refractory anal fistulas healed completely within 4 weeks of onset of therapy, and CD4 T cell counts increased from 220 to 509 cells/μl after 2 years of treatment (1/17/17), demonstrating the effectiveness of this regimen in achieving clinical improvement.

**Fig. 2:**
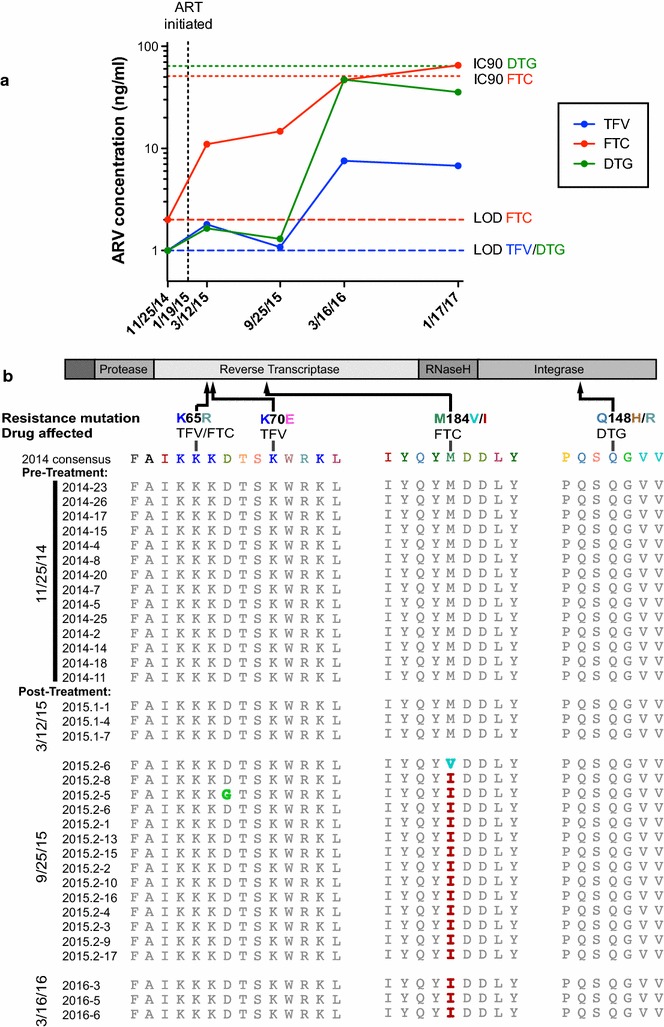
Plasma drug concentrations and emergence of FTC resistance. **a** Plasma concentrations of tenofovir (TFV, *blue*), emtricitabine (FTC, *red*), and dolutegravir (DTG, *green*) are shown for one pre-treatment (11/25/14) and four post-treatment samples (3/12/15, 9/25/15, 3/16/16, 1/17/17; viral loads corresponding to these time points are depicted in Fig. 1b). Limits of detection (LOD) for FTC and TFV/DTG are shown as *red* and *blue dashed lines*, respectively. The in vitro 90% HIV-1 inhibitory concentrations (IC_90_) for FTC and DTG are shown in *red* and *green dotted lines*, respectively. The in vitro 50% HIV-1 inhibitory concentrations (IC_50_) of TFV (7.8 μg/ml) is off-scale and thus not shown. **b** Mutations in reverse transcriptase and integrase regions associated with TFV, FTC, and DTG drug resistance in HIV-1 infection. A schematic representation of *pol* gene proteins is shown with common amino acid substitutions known to confer drug resistance indicated. *Pol* sequences generated from Cotton’s plasma using SGA approaches are listed by time points. Residues that are mutated compared to the pre-treatment consensus sequence are highlighted in *color* (identical residues are shown in *grey*). *Pol* sequences were also analyzed for less common substitutions associated with DTG resistance [54], but were free of such mutations

Cotton received ART daily by oral administration of crushed tablets dissolved in diluted fruit syrup, but caretakers noticed that he did not always ingest his medication completely. We thus continued to monitor his virus load in blood samples obtained during his bi-annual medical evaluations. Indeed, nine months following initiation of therapy (9/25/15), virus was again detectable (4760 RNA copies/ml) in his plasma (Fig. 1b). To determine whether this rebound viremia was due to insufficient drug levels, one pre-treatment and four post-treatment plasma samples were sent to the Clinical Pharmacology and Analytical Chemistry Core at the UNC Center for AIDS Research to quantitate plasma concentrations. This analysis revealed drug concentrations of 1.08, 14.70 and 1.30 ng/ml for tenofovir (TFV, the metabolized product of TDF), FTC and DTG, respectively, at the first time point of viral rebound (9/25/15) (Fig. 2a), which were significantly below the in vitro 50% HIV-1 inhibitory concentration (IC_50_) of TFV (7.8 μg/ml) [29] and the 90% HIV-1 inhibitory concentrations (IC_90_) of FTC (51 ng/ml) and DTG (64 ng/ml) [30, 31], respectively. After caretakers improved oral administration by suspending the crushed tablets in more concentrated fruit syrup, plasma concentrations of TFV, FTC and DTG increased to 7.56, 46.90 and 47.20 ng/ml, respectively (Fig. 2a), which resulted in a concomitant reduction in viremia back to undetectable levels (Fig. 1b). However, these plasma concentrations were still significantly lower than typical human trough concentrations for this same regimen (54 ng/ml for TFV, 75 ng/ml for FTC and 830 ng/ml for DTG) [32, 33]. Since FTC and TDF are metabolized in the kidney [34], while DTG is metabolized in the liver [35], the uniformly low plasma concentrations are most likely due to insufficient drug uptake, although differences in pharmacokinetics between chimpanzees and humans cannot be excluded. The timing of sample collection could have also played a role since the interval between plasma collection and the last drug administration was not recorded.

## Development of drug resistance

Insufficient drug concentrations are known to facilitate the emergence of drug resistance [36]. We thus used single genome amplification (SGA) to generate full-length *pol* gene sequences from pre- and post-treatment plasma samples. Briefly, plasma RNA was extracted, reverse transcribed, and the resulting cDNA end-point diluted to single template levels. Full-length *pol* genes were amplified and sequenced, and amplicons comprising more than one variant discarded [37]. This analysis revealed the emergence of M184V and M184I mutations in the conserved YMDD motif of the viral reverse transcriptase (RT) nine months after the onset of therapy (Fig. 2b). In HIV-1 infected humans, these mutations are known to confer resistance to FTC and to represent a first indication of a failing ART regimen [36, 38]. Viruses containing the M184I (ATG to ATA) mutation usually emerge first, but are rapidly outcompeted by viruses containing the M184V (ATG to GTG) mutation [39] due to their enhanced replication fitness [40]. Interestingly, this was not the case in Cotton where both M184I (ATG to ATA) and M184V (ATG to GTA) were observed at viral rebound, but M184I remained the predominant mutation (Fig. 2b). Importantly, other mutations known to be associated with TFV and DVG resistance were not observed in any of the post-treatment sequences, and the most recent plasma sample, which was RT-qPCR negative (1/17/17), also failed to yield *pol* amplicons, suggesting complete virus suppression.

**Fig. 3:**
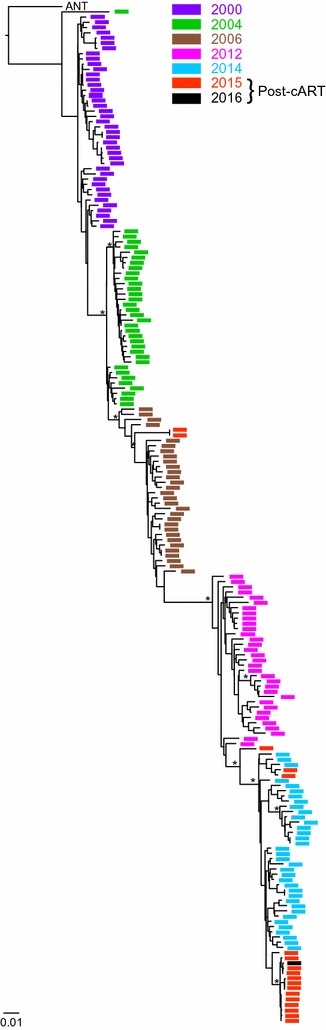
SIVcpzANT *env* gene evolution over time. A maximum likelihood tree depicting the phylogenetic relationships of full-length *env* nucleotide sequences generated from Cotton’s plasma using SGA approaches is shown for a 16-year time period. Samples are *colored* by time point (11/30/2000, *purple*; 7/19/2004, *green*; 11/01/2006, *brown*; 9/25/2012, *magenta*; 11/25/2014, *blue*; 9/25/2015, *red*; 3/16/2016, *black*), with sequences from 2015 and 2016 representing rebound virus after insufficient suppression of viral replication due to low drug concentrations. Sequences were analyzed using PhyML [47] based on an evolutionary substitution model (TVM+I+G) selected by jModelTest [55]. The tree is rooted using the original SIVcpzANT sequence (U42720). Bootstrap values (of 1000 replicates) ≥80% are shown for major clades. The *scale bar* indicates 0.01 substitutions per site

The low plasma drug concentrations and emergence of FTC escape mutations suggested that the initial treatment failed to fully suppress virus replication in Cotton, despite viral load measurements below the limits of detection (<200 copies/ml) in the first post-treatment sample (3/12/15). Treatment interruptions of fully suppressed HIV-1 infected humans usually result in the emergence of multiple viral lineages [41–44] that arise stochastically from latently infected cells rather than from concurrent low-level replication [45]. To examine the origin of Cotton’s rebound virus, we used SGA to amplify 3′ half viral genomes before and after treatment initiation [46]. Maximum likelihood analysis [47] of 194 full-length *env* gene sequences revealed that the majority of rebound viruses fell within the radiation of strains sampled immediately prior to treatment (11/25/14), with only two post-treatment sequences clustering with viruses from 2006 (Fig. 3). Thus, viral rebound in Cotton resulted primarily from an outgrowth of low-level viremia rather than from reactivated latently infected cells. Our ability to amplify three *pol* gene sequences from the first post treatment sample (3/12/15) that was RT-qPCR negative is consistent with this (Fig. 2b). We also observed progressive diversification of pre-treatment SIVcpzANT *env* sequences over time, similar to what has been described for HIV-1 infected humans [48, 49]. Although these *env* sequences clustered almost exclusively by time point, this exaggerated bottlenecking is likely due to long sampling intervals rather than differences in the pathways of SIVcpz and HIV-1 diversification. To examine this further, we analyzed the C2-V5 region, which has previously been shown to diversify at a rate of approximately 1% per year in HIV-1 infected humans [49]. Using BEAST [50] to estimate the rate of SIVcpzANT diversification in the same C2-V5 region, we found a nearly identical substitution rate of 1.01% per year. Since the earlier report [49] had not used SGA to generate viral sequences nor BEAST for their analysis, we reexamined one subject whose viral sequences were endpoint diluted prior to PCR amplification. This analysis yielded a substitution rate of 1.08% per year. Thus, the rate of SIVcpz evolution in chimpanzees appears to be very similar to that of HIV-1 in humans.

## Conclusions

In summary, we report here the first case of progressive immunodeficiency and clinical disease in a western chimpanzee experimentally infected with a naturally occurring strain of SIVcpz*Pts*. While Cotton’s laboratory values did not meet the formal definition of AIDS (<200 CD4+ T cells/μl), his low CD4+ T cell counts, high plasma viral loads, treatment refractory soft tissue infections, and marked clinical improvement following reduction of plasma viremia by antiretroviral therapy indicates that SIVcpz caused his immunodeficiency. These results thus dispel the notion that SIVcpz is non-pathogenic in captive environments, or that SIVcpzANT is an attenuated strain [17]. They also show that SIVcpz is pathogenic in chimpanzees that are not naturally infected with this virus. In addition to Cotton, two other experimentally SIVcpz infected *P. t. verus* chimpanzees are still alive. One (X176) was reported to have a CD4+ T cell count of 115 cells/μl, which represents an AIDS-defining criterion, while the other (X284) maintained low viral loads and normal CD4 counts 16 years after infection [17]. The average time to AIDS progression in untreated HIV-1 infected humans has been estimated to range between 10 and 15 years. Thus, the natural history of SIVcpz infection, including the rate of disease progression, likely differs from that of HIV-1 infected humans, although the number of long-term followed apes is too small to draw definitive conclusions.

We also show that SIVcpz associated immunodeficiency can be effectively treated with antiretroviral therapy, which decreased plasma viremia in Cotton to undetectable levels within 7 weeks and increased CD4+ T cell counts to above 500 cells/μl within 2 years after onset of therapy, although low plasma drug concentrations resulted in the emergence of FTC resistance mutations. A considerable number of HIV-1 and SIVcpz infected chimpanzees are still housed in US primate centers. This study, together with previous reports of disease progression in HIV-1 infected captive chimpanzees [51–53], indicates that animals with high viremia and reduced CD4+ T cell counts are at risk of developing AIDS and should be treated to prevent clinical disease. However, difficulties in maintaining strict adherence of daily oral administration and possible chimpanzee-specific differences in pharmacokinetics will require careful monitoring of drug concentrations and virus suppression. This is also true for Cotton, who currently has undetectable viral loads, but whose low plasma concentrations of TFV and DFV place him at high risk of developing further resistance and treatment failure.

## Abbreviations

SIVcpz: simian immunodeficiency virus of chimpanzees
HIV-1: human immunodeficiency virus type 1
AIDS: acquired immunodeficiency syndrome
SGA: single genome amplification
ART: antiretroviral therapy
DTG: dolutegravir
TDF: tenofovir disoproxil fumarate
TFV: tenofovir
FTC: emtricitabine
SIVmac: simian immunodeficiency virus of macaques

## References

[1] Keele BF, Van Heuverswyn F, Li Y, Bailes E, Takehisa J, Santiago ML (2006). Chimpanzee reservoirs of pandemic and nonpandemic HIV-1. Science.

[2] Santiago ML, Lukasik M, Kamenya S, Li Y, Bibollet-Ruche F, Bailes E (2003). Foci of endemic simian immunodeficiency virus infection in wild-living eastern chimpanzees (*Pan troglodytes schweinfurthii*). J Virol.

[3] Santiago ML, Rodenburg CM, Kamenya S, Bibollet-Ruche F, Gao F, Bailes E (2002). SIVcpz in wild chimpanzees. Science.

[4] Van Heuverswyn F, Li Y, Bailes E, Neel C, Lafay B, Keele BF (2007). Genetic diversity and phylogeographic clustering of SIVcpzPtt in wild chimpanzees in Cameroon. Virology.

[5] Li Y, Ndjango JB, Learn GH, Ramirez MA, Keele BF, Bibollet-Ruche F (2012). Eastern chimpanzees, but not bonobos, represent a simian immunodeficiency virus reservoir. J Virol.

[6] Switzer WM, Parekh B, Shanmugam V, Bhullar V, Phillips S, Ely JJ (2005). The epidemiology of simian immunodeficiency virus infection in a large number of wild- and captive-born chimpanzees: evidence for a recent introduction following chimpanzee divergence. AIDS Res Hum Retroviruses.

[7] Prince AM, Brotman B, Lee D-H, Andrus L, Valinsky J, Marx P (2002). Lack of evidence for HIV type 1-related SIVcpz infection in captive and wild chimpanzees (*Pan troglodytes verus*) in West Africa. AIDS Res Hum Retroviruses.

[8] Bailes E, Gao F, Bibollet-Ruche F, Courgnaud V, Peeters M, Marx PA (2003). Hybrid origin of SIV in chimpanzees. Science.

[9] Keele BF, Jones JH, Terio KA, Estes JD, Rudicell RS, Wilson ML (2009). Increased mortality and AIDS-like immunopathology in wild chimpanzees infected with SIVcpz. Nature.

[10] Terio KA, Kinsel MJ, Raphael J, Mlengeya T, Lipende I, Kirchhoff CA (2011). Pathologic lesions in chimpanzees (*Pan trogylodytes schweinfurthii*) from Gombe National Park, Tanzania, 2004–2010. J Zoo Wildl Med.

[11] Rudicell RS, Holland Jones J, Wroblewski EE, Learn GH, Li Y, Robertson JD (2010). Impact of simian immunodeficiency virus infection on chimpanzee population dynamics. PLoS Pathog.

[12] Ely JJ, Dye B, Frels WI, Fritz J, Gagneux P, Khun HH (2005). Subspecies composition and founder contribution of the captive U.S. chimpanzee (*Pan troglodytes*) population. Am J Primatol.

[13] Etienne L, Nerrienet E, LeBreton M, Bibila GT, Foupouapouognigni Y, Rousset D (2011). Characterization of a new simian immunodeficiency virus strain in a naturally infected *Pan troglodytes troglodytes* chimpanzee with AIDS related symptoms. Retrovirology.

[14] Gilden R, Arthur L, Robey W, Kelliher J, Graham C, Fischinger P (1986). HTLV-III antibody in a breeding chimpanzee not experimentally exposed to the virus. Lancet.

[15] Peeters M, Honare C, Huet T, Bedjabaga L, Ossari S, Bussi P (1989). Isolation and partial characterization of an HIV-related virus occurring naturally in chimpanzees in Gabon. AIDS.

[16] Nerrienet E, Santiago ML, Foupouapouognigni Y, Bailes E, Mundy NI, Njinku B (2005). Simian immunodeficiency virus infection in wild-caught chimpanzees from Cameroon. J Virol.

[17] Greenwood EJD, Schmidt F, Kondova I, Niphuis H, Hodara VL, Clissold L (2015). Simian immunodeficiency virus infection of chimpanzees (*Pan troglodytes*) shares features of both pathogenic and non-pathogenic lentiviral infections. PLoS Pathog.

[18] Heeney JL, Rutjens E, Verschoor EJ, Niphuis H, ten Haaft P, Rouse S (2006). Transmission of simian immunodeficiency virus SIVcpz and the evolution of infection in the presence and absence of concurrent human immunodeficiency virus type 1 infection in chimpanzees. J Virol.

[19] Peeters M, Fransen K, Delaporte E, Van den Haesevelde M, Gershy-Damet GM, Kestens L (1992). Isolation and characterization of a new chimpanzee lentivirus (simian immunodeficiency virus isolate cpz-ant) from a wild-captured chimpanzee. AIDS..

[20] Ihrig M, Tassinary LG, Bernacky B, Keeling ME (2001). Hematologic and serum biochemical reference intervals for the chimpanzee (*Pan troglodytes*) categorized by age and sex. Comp Med.

[21] Etienne L, Eymard-Duvernay S, Aghokeng A, Butel C, Monleau M, Peeters M (2013). Single real-time reverse transcription-PCR assay for detection and quantification of genetically diverse HIV-1, SIVcpz, and SIVgor strains. J Clin Microbiol.

[22] Vanden Haesevelde MM, Peeters M, Jannes G, Janssens W, van der Groen G, Sharp P (1996). Sequence analysis of a highly divergent HIV-1-related lentivirus isolated from a wild captured chimpanzee. Virology.

[23] Panel on antiretroviral guidelines for adults and adolescents. Guidelines for the use of antiretroviral agents in HIV-1-infected adults and adolescents. Department of Health and Human Services. http://www.aidsinfo.nih.gov/ContentFiles/AdultandAdolescentGL.pdf. Accessed Jan 2017 (page F-3).

[24] Treviño A, Cabezas T, Lozano AB, García-Delgado R, Force L, Fernández-Montero JM (2015). Dolutegravir for the treatment of HIV-2 infection. J Clin Virol.

[25] Saravolatz LD, Saag MS (2006). Emtricitabine, a new antiretroviral agent with activity against HIV and hepatitis B virus. Clin Infect Dis.

[26] Raffi F, Rachlis A, Stellbrink H-J, Hardy WD, Torti C, Orkin C (2013). Once-daily dolutegravir versus raltegravir in antiretroviral-naive adults with HIV-1 infection: 48 week results from the randomised, double-blind, non-inferiority SPRING-2 study. Lancet.

[27] Schooley RT, Ruane P, Myers RA, Beall G, Lampiris H, Berger D (2002). Tenofovir DF in antiretroviral-experienced patients: results from a 48-week, randomized, double-blind study. AIDS.

[28] Del Prete GQ, Smedley J, Macallister R, Jones GS, Li B, Hattersley J (2015). Short Communication: comparative evaluation of coformulated injectable combination antiretroviral therapy regimens in simian immunodeficiency virus-infected rhesus macaques. AIDS Res Hum Retroviruses.

[29] Mesquita PMM, Rastogi R, Segarra TJ, Teller RS, Torres NM, Huber AM (2012). Intravaginal ring delivery of tenofovir disoproxil fumarate for prevention of HIV and herpes simplex virus infection. J Antimicrob Chemother.

[30] Cottrell ML, Hadzic T, Kashuba ADM (2013). Clinical pharmacokinetic, pharmacodynamic and drug-interaction profile of the integrase inhibitor dolutegravir. Clin Pharmacokinet.

[31] Flynn PM, Mirochnick M, Shapiro DE, Bardeguez A, Rodman J, Robbins B (2011). Pharmacokinetics and safety of single-dose tenofovir disoproxil fumarate and emtricitabine in HIV-1-infected pregnant women and their infants. Antimicrob Agents Chemother.

[32] Min S, Sloan L, DeJesus E, Hawkins T, McCurdy L, Song I (2011). Antiviral activity, safety, and pharmacokinetics/pharmacodynamics of dolutegravir as 10-day monotherapy in HIV-1-infected adults. AIDS.

[33] Blum MR, Chittick GE, Begley JA, Zong J (2007). Steady-state pharmacokinetics of emtricitabine and tenofovir disoproxil fumarate administered alone and in combination in healthy volunteers. J Clin Pharmacol.

[34] Dando TM, Wagstaff AJ (2004). Emtricitabine/tenofovir disoproxil fumarate. Drugs.

[35] Min S, Song I, Borland J, Chen S, Lou Y, Fujiwara T (2010). Pharmacokinetics and safety of S/GSK1349572, a next-generation HIV integrase inhibitor, in healthy volunteers. Antimicrob Agents Chemother.

[36] Clavel F, Hance AJ (2004). HIV drug resistance. New Engl J Med.

[37] Salazar-Gonzalez JF, Bailes E, Pham KT, Salazar MG, Guffey MB, Keele BF (2008). Deciphering human immunodeficiency virus type 1 transmission and early envelope diversification by single-genome amplification and sequencing. J Virol.

[38] Nelson M, Schiavone M (2004). Emtricitabine (FTC) for the treatment of HIV infection. Int J Clin Pract.

[39] Schuurman R, Nijhuis M, Rv Leeuwen, Schipper P, Jong Dd, Collis P (1995). Rapid changes in human immunodeficiency virus type 1 RNA load and appearance of drug-resistant virus populations in persons treated with lamivudine (3TC). J Infect Dis.

[40] Back NK, Nijhuis M, Keulen W, Boucher CA, Oude Essink BO, van Kuilenburg AB (1996). Reduced replication of 3TC-resistant HIV-1 variants in primary cells due to a processivity defect of the reverse transcriptase enzyme. EMBO J.

[41] Rothenberger MK, Keele BF, Wietgrefe SW, Fletcher CV, Beilman GJ, Chipman JG (2015). Large number of rebounding/founder HIV variants emerge from multifocal infection in lymphatic tissues after treatment interruption. Proc Natl Acad Sci USA..

[42] Kearney MF, Wiegand A, Shao W, Coffin JM, Mellors JW, Lederman M (2016). Origin of rebound plasma HIV includes cells with identical proviruses that are transcriptionally active before stopping of antiretroviral therapy. J Virol.

[43] Bednar MM, Hauser BM, Zhou S, Jacobson JM, Eron JJ, Frank I (2016). Diversity and tropism of HIV-1 rebound virus populations in plasma level after treatment discontinuation. J Infect Dis.

[44] Bar KJ, Sneller MC, Harrison LJ, Justement JS, Overton ET, Petrone ME (2016). Effect of HIV antibody VRC01 on viral rebound after treatment interruption. New Engl J Med.

[45] Joos B, Fischer M, Kuster H, Pillai SK, Wong JK, Böni J (2008). HIV rebounds from latently infected cells, rather than from continuing low-level replication. Proc Natl Acad Sci USA.

[46] Li H, Bar KJ, Wang S, Decker JM, Chen Y, Sun C (2010). High multiplicity infection by HIV-1 in men who have sex with men. PLoS Pathog.

[47] Guindon S, Dufayard J-F, Lefort V, Anisimova M, Hordijk W, Gascuel O (2010). New algorithms and methods to estimate maximum-likelihood phylogenies: assessing the performance of PhyML 3.0. Syst Biol.

[48] Zanini F, Brodin J, Thebo L, Lanz C, Bratt G, Albert J (2015). Population genomics of intrapatient HIV-1 evolution. eLife.

[49] Shankarappa R, Margolick JB, Gange SJ, Rodrigo AG, Upchurch D, Farzadegan H (1999). Consistent viral evolutionary changes associated with the progression of human immunodeficiency virus type 1 infection. J Virol.

[50] Drummond AJ, Rambaut A (2007). BEAST: Bayesian evolutionary analysis by sampling trees. BMC Evol Biol.

[51] Novembre FJ, de Rosayro J, Nidtha S, O’Neil SP, Gibson TR, Evans-Strickfaden T (2001). Rapid CD4+ T-cell loss induced by human immunodeficiency virus type 1NC in uninfected and previously infected chimpanzees. J Virol.

[52] Novembre FJ, Saucier M, Anderson DC, Klumpp SA, O’Neil SP, Brown CR (1997). Development of AIDS in a chimpanzee infected with human immunodeficiency virus type 1. J Virol.

[53] O’Neil SP, Novembre FJ, Hill AB, Suwyn C, Hart CE, Evans-Strickfaden T (2000). Progressive infection in a subset of HIV-1-positive chimpanzees. J Infect Dis.

[54] Wensing AM, Calvez V, Gunthard HF, Johnson VA, Paredes R, Pillay D (2017). 2017 update of the drug resistance mutations in HIV-1. Top Antivir Med.

[55] Darriba D, Taboada GL, Doallo R, Posada D (2012). jModelTest 2: more models, new heuristics and high-performance computing. Nat Methods.

